# Ultrafast processes triggered by one- and two-photon excitation of a photochromic and luminescent hydrazone

**DOI:** 10.3762/bjoc.15.236

**Published:** 2019-10-15

**Authors:** Alessandro Iagatti, Baihao Shao, Alberto Credi, Barbara Ventura, Ivan Aprahamian, Mariangela Di Donato

**Affiliations:** 1 LENS – European Laboratory for Non-linear Spectroscopy, via N. Carrara 1, 50019 Sesto Fiorentino (FI), Italy; 2 Department of Chemistry, Dartmouth College, Hanover, New Hampshire 03755, United States; 3 CLAN – Center for Light Activated Nanostructures, Dipartimento di Scienze e Tecnologie Agro-alimentari, Università di Bologna, viale Fanin 50, 40127 Bologna, Italy; 4 Istituto per la Sintesi Organica e la Fotoreattività, Consiglio Nazionale delle Ricerche, via Gobetti 101, 40129 Bologna, Italy; 5 INO – Istituto Nazionale di Ottica, Largo Enrico Fermi 6, 50125 Firenze, Italy

**Keywords:** hydrazone, molecular switch, pump-probe spectroscopy, time-resolved fluorescence

## Abstract

In this work we apply a combination of steady state and time resolved luminescence and absorption spectroscopies to investigate the excited-state dynamics of a recently developed molecular photoswitch, belonging to the hydrazone family. The outstanding properties of this molecule, involving fluorescence toggling, bistability, high isomerization quantum yield and non-negligible two-photon absorption cross section, make it very promising for numerous applications. Here we show that the light induced *Z/E* isomerization occurs on a fast <1 ps timescale in both toluene and acetonitrile, while the excited state lifetime of the *Z*-form depends on solvent polarity, suggesting a partial charge transfer nature of its low lying excited state. Time-resolved luminescence measurements evidence the presence of a main emission component in the 500–520 nm spectral range, attributed to the *Z-*isomer, and a very short living blue-shifted emission, attributed to the *E-*isomer. Finally, transient absorption measurements performed upon far-red excitation are employed as an alternative method to determine the two-photon absorption cross-section of the molecule.

## Introduction

Molecular switches are systems that are able to rapidly respond to an external stimulus, which can be of chemical or physical nature, through a variation of their conformational, chemical or physical properties [1]. The possibility to control their operation in a direct and specific manner paves the way for applications in many different fields, involving the production of responsive materials and surfaces [2–3], energy conversion [4–6], catalysis [7], drug delivery [8–9], design of molecular machines [10–13], super resolution microscopy [14–15], together with biological applications, among which photopharmacology is currently gaining increasing attention [16–19].

Photochromic molecules, which respond to light as an external stimulus, raise particular interest among the different classes of switches which have been developed to date. Light enables very specific spatial and temporal control of the switching event, allowing for selective response and bidirectional operation. It is thus not surprising that different classes of photoswitches have been developed and successfully employed in many technological fields [20–23]. Among others, azobenzenes [24], spiropyranes [25], diarylethenes [26] and their derivatives have been intensively applied. Despite the numerous successful applications of several synthetized switches, some drawbacks still remain, calling for the development of new systems with improved behavior. Although the properties of a successful photoswitch have to be tailored on the application for which it is designed, there are several aspects whose improvement can be of benefit on a general basis. Major concerns on a widespread use of the most commonly employed systems have indeed often be related to low quantum yields or poor photochemical stability, low fatigue resistance, or difficult synthesis. Furthermore, in the specific case of biological applications, inappropriate absorption wavelength is often an issue, considering that most of the commonly used switches absorb in the UV spectral window, as well as low solubility in water.

Among the variety of newly developed systems, a promising class of switches is based on the hydrazone molecular motif [27]. These systems present a variety of interesting properties: they can be chemically or photochemically controlled, can undergo a configurational change by acid or base addition and have interconversion timescales which can span over several orders of magnitude. Furthermore, a substantial shift of their absorption profile can be enabled through substitution patterns, yielding systems that absorb in the red part of the visible spectrum [28].

Recently, a new hydrazone-based photochromic compound, exhibiting outstanding properties, has been synthetized and characterized [29]. This molecule presents fluorescence ON/OFF switching under both one-photon and two-photon excitation (i.e., near infrared (NIR) light), which is also maintained in serum and solid state, has a very high photochemical stability and excellent fatigue resistance. Although the main photochemical properties of this molecule have been recently reported [29], a detailed analysis of its photoswitching mechanism, aimed at characterizing the timescale of the photoinduced structure variation and the solvent dependence of its fluorescence properties, is still lacking. Here we present a spectroscopic characterization of this hydrazone species, using both steady state and time resolved absorption and fluorescence techniques with the aim of better characterizing the actinic step of its operation. Furthermore, we employ time resolved spectroscopy to evaluate the two-photon absorption cross section of the molecule and, by comparing the results with those previously obtained using a fluorescence method, we show that this can be a successful alternative to evaluate two-photon properties, particularly useful in case of non-fluorescent molecules.

## Results and Discussion

### Spectroscopic properties

**Figure 1:**
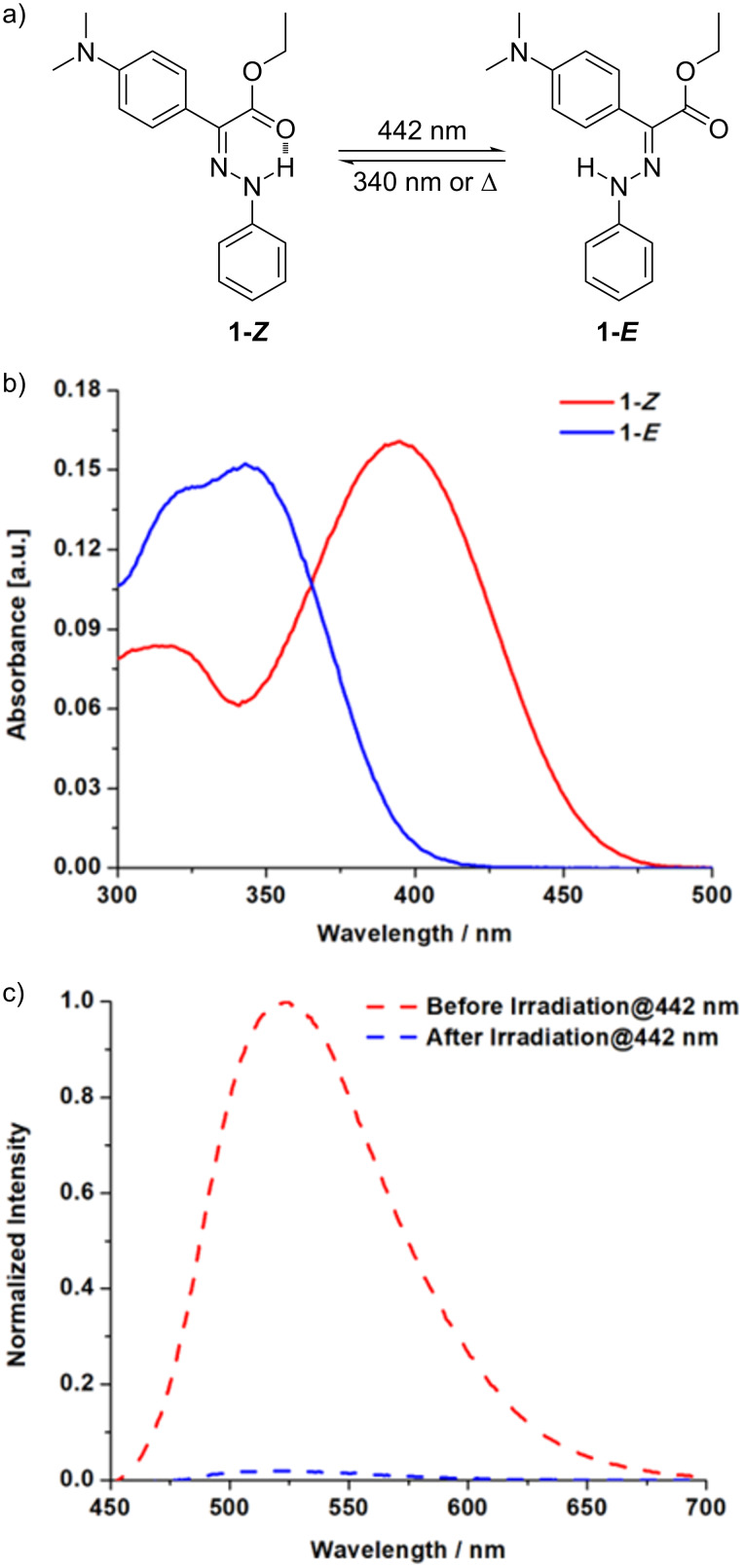
a) The photoinduced *Z*/*E* isomerization of hydrazone **1**, and accompanied changes in b) UV–vis absorption (1 × 10^−5^ M; toluene) and c) fluorescence emission spectra (1 × 10^−6^ M; toluene) before (red) and after (blue) irradiation at 442 nm.

The UV–vis absorption spectrum of hydrazone **1** shows an intense absorption band peaked at 395 nm in toluene, as shown in Figure 1. Irradiation of a solution of **1** using 442 nm light induces a *Z/E* isomerization resulting in a color change, evidenced by the decrease of the absorption at 395 nm and the appearance of a new band with a maximum at 343 nm.

The *E*-form is extremely stable (half-life of 75 years in toluene at room temperature) and can be reverted to the *Z*-form by irradiation at 340 nm or heat. The molecule also presents peculiar fluorescence properties. Indeed, while the *Z*-form has an intense emission band peaked at 525 nm in toluene, fluorescence is suppressed for the *E*-form (Figure 1). The maximum of the fluorescence band and the emission quantum yield depend on the solvent, with the emission strongly quenched in protic media [29].

### Time-resolved fluorescence

Detailed analysis of the fluorescence features of the two forms in toluene has been performed by time-resolved luminescence measurements in the picosecond time regime (see Materials and Methods for details).

**Figure 2:**
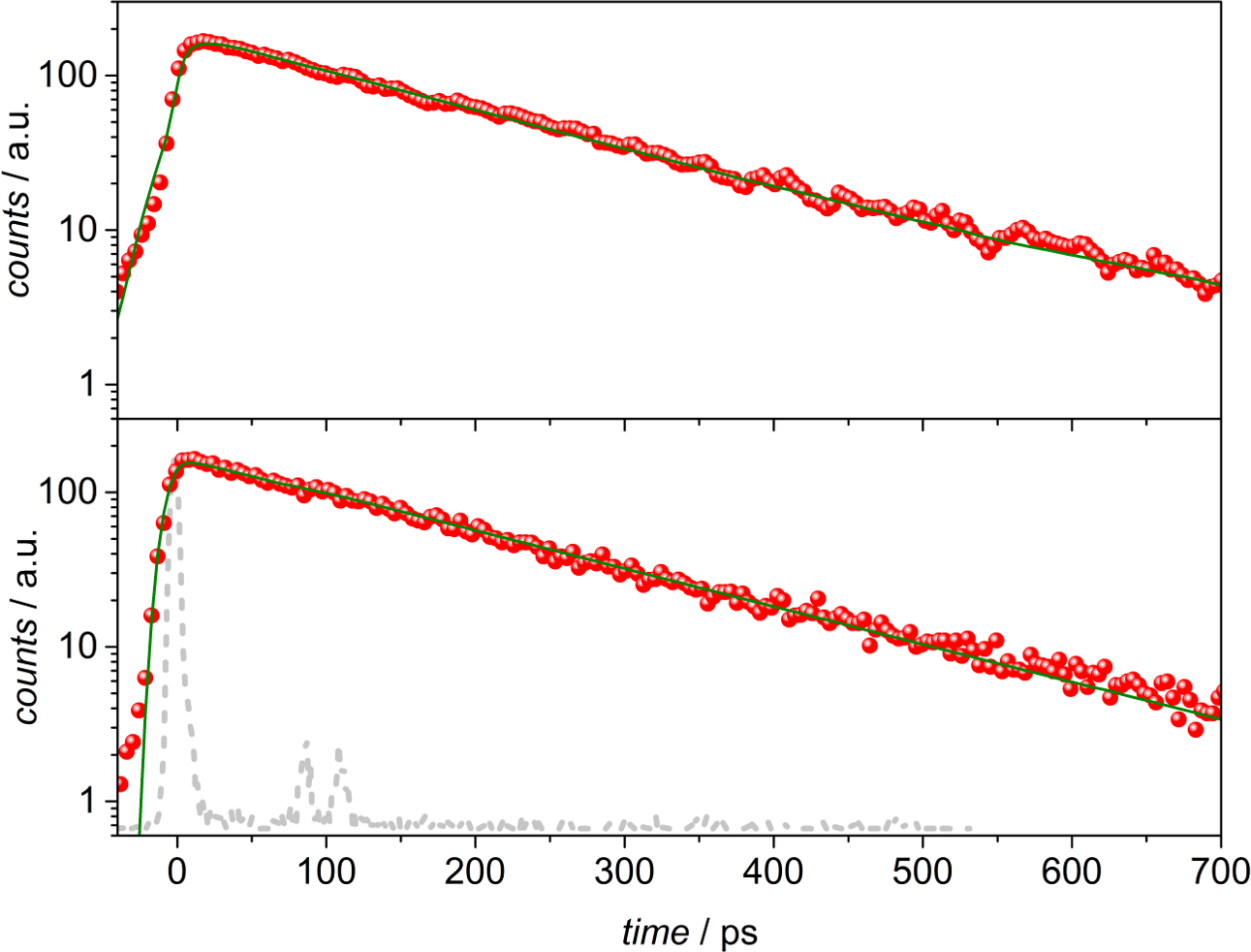
Fluorescence decays (dots) in the 500–520 nm spectral region (emission range of the *Z*-isomer) for **1** in toluene upon excitation of the *E*-form at 350 nm (top; induced *Z*-emission), and upon excitation of the *Z*-form at 400 nm (bottom). The mono-exponential fittings are reported as full lines. The excitation profile is shown in dashed light grey.

Upon excitation of **1** in the *Z* form at 400 nm, the fluorescence is characterized by a mono-exponential decay on the whole emission spectral range, with an average lifetime of 173 ps (Figure 2, bottom). It can be pointed out that the fluorescence intensity gradually decreases during the measurement, because of the photoisomerisation process, but the use of a low laser power (0.8 µJ/pulse) and the acquisition of a large number of frames (10000) in photon counting mode allowed the characterization of the emission properties of this form. The obtained lifetime value is in accordance with the excited state lifetime previously measured for the *Z*-isomer [29].

**Figure 3:**
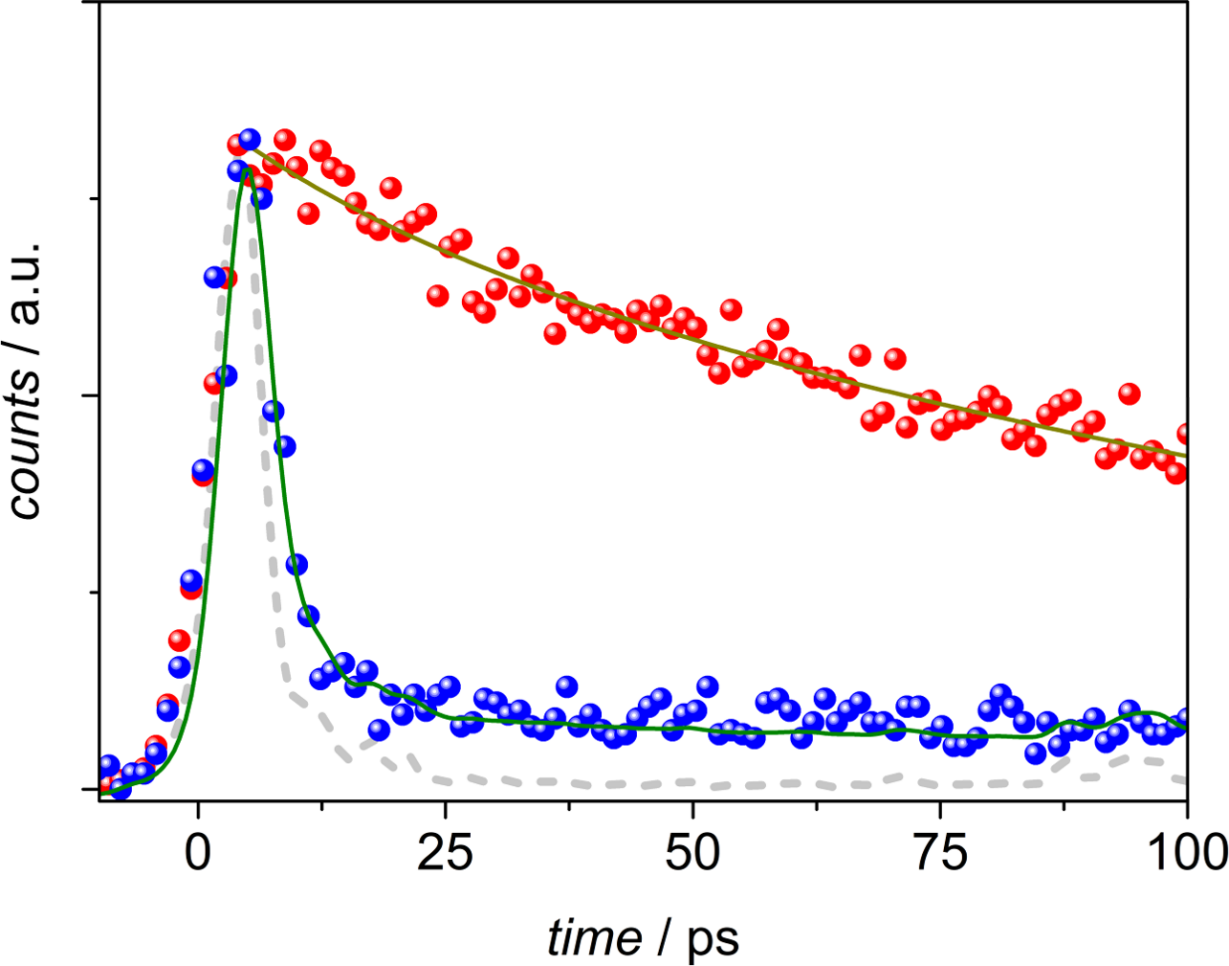
Fluorescence decays (dots) in the 500–520 nm spectral region (red; induced *Z*-emission), and in the 440–460 nm spectral region (blue) for **1** in toluene upon excitation of the *E*-form at 350 nm. The fittings are reported as full lines. The excitation profile is shown in dashed light grey.

After complete *Z/E* isomerization, obtained by irradiating the solution at 440 nm, the time-resolved luminescence of the *E*-form was analyzed upon excitation at 350 nm. The spectral distribution of the excited-state decays reveals the presence of a short component in the blue region of the spectrum (440–460 nm) which is not present in the remaining emission region (500–600 nm). A weak emission in this region is also observed in the steady-state fluorescence spectra obtained upon excitation, below 390 nm, of an *E*-rich acetonitrile solution of **1**, which might be attributed to the emission of this isomer as the more intense band at 570 nm originates from the *Z*-form (Figure S1, Supporting Information File 1). Figure 3 shows the comparison between the luminescence decays collected in the 440–460 nm range (blue dots) and in the 500–520 nm range (red dots) of the same streak camera image. A double exponential fitting of the profile in the blue region results in a main component with a lifetime of 1.3 ps (at the limit of the detection of the system: 1.0 ps) accounting for 97% of the decay, and a second component with a longer lifetime of ca. 160 ps. The decay in the 500–520 nm region can be fitted by a mono-exponential function yielding a lifetime of 160 ps, close to the value obtained for the fluorescence of the Z-form upon excitation at 400 nm (the comparison on a longer time scale is shown in Figure 2). The short lifetime of 1.3 ps can be ascribed to the fluorescence of the *E*-form, mainly centered in the 440–460 nm region, which accounts for the very low emission quantum yield of this form observed in the steady-state experiment. The emission from the *Z*-form is detected even upon excitation at 350 nm, because of the induced *E*/*Z* photoisomerization process occurring within the experiment.

Finally, the kinetics of the fluorescence decay of the *Z*-isomer has also been determined in acetonitrile, upon excitation at 400 nm, resulting in a lifetime of 479 ps (Figure S2, Supporting Information File 1).

### Transient absorption spectroscopy

**Figure 4:**
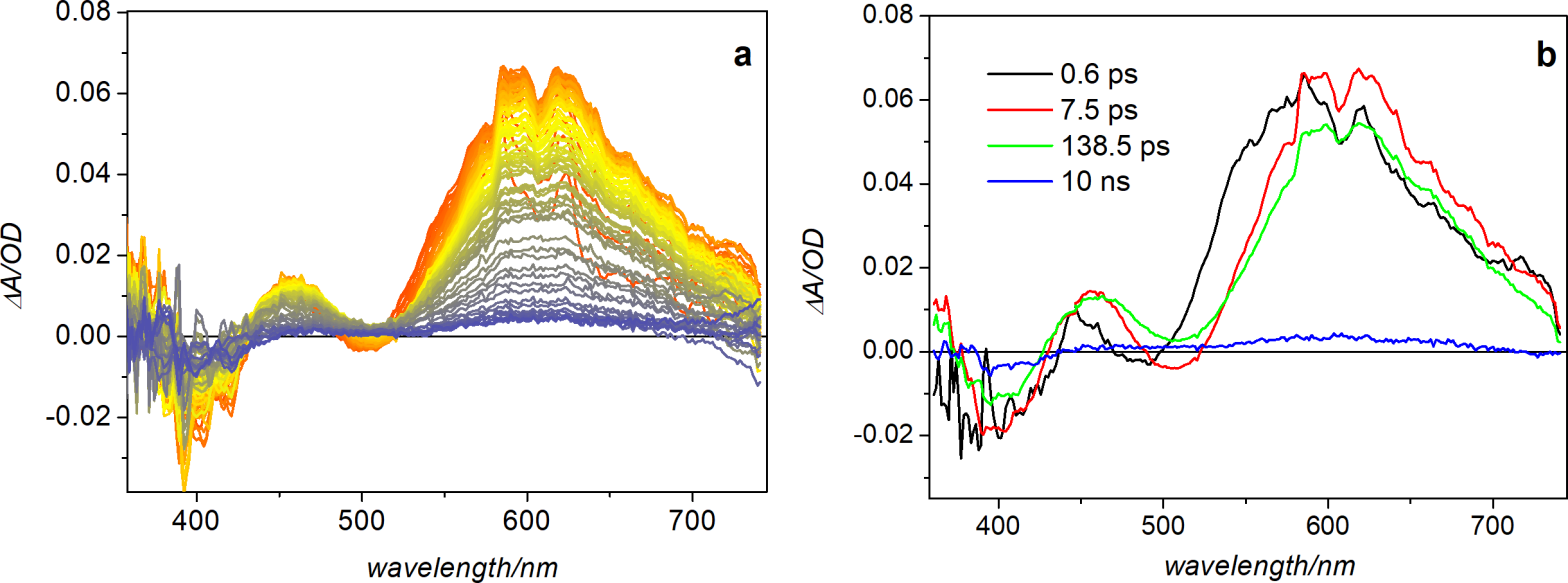
a) Transient absorption data recorded for hydrazone **1** in toluene upon excitation at 400 nm; b) EADS (evolution associated difference spectra) obtained by global fit of the data.

To get more insights into the isomerization and fluorescence mechanism of **1**, we measured transient absorption spectra of the molecule in different solvents. A fresh solution of the *Z*-form was excited using 400 nm light, and spectra were recorded in a time interval spanning from a few hundred femtoseconds up to 1.5 ns. The transient signal recorded for a solution of **1** dissolved in toluene shows the appearance of a negative band peaking at about 400 nm, corresponding to the bleaching of the ground state absorption, an intense very broad positive excited state absorption band peaking at about 600 nm and a less intense positive band in the low wavelength region. A pronounced dip is furthermore observed at ca. 500 nm, most probably because of the superposition of a stimulated emission signal with the broad excited state absorption band, see Figure 4a. The excited state absorption signal increases in intensity and broadens towards the red on a fast timescale. At the same time the stimulated emission band partially recovers, shifting towards the red and a positive band in the lower wavelength region increases in intensity. The rise of a positive signal at <380 nm, where absorption of the *E*-isomer is expected, signals the occurrence of isomerization. This event is also associated to the aforementioned evolution in the visible region, clearly indicating a variation of the excited state electronic distribution. No further band-shape changes are observable at a later timescale, the transient signal almost completely recovers on a ca. 140 ps timescale, which is similar to the fluorescence lifetime of the *Z*-isomer measured in this solvent.

To extract a quantitative kinetic information from the transient absorption data, they have been fitted using a global analysis procedure, which consists in the simultaneous fit of the kinetic traces in the entire probed spectral window with a combination of exponential decay functions [30]. The number of exponential components is determined by performing a preliminary SVD (singular value decomposition) analysis of the kinetic traces matrix [31]. In this case, although three exponential functions could be sufficient to satisfactory fit the data, the addition of a fourth long living component, associated to a small spectral offset remaining on the long timescale, substantially improved the fit. The output of the global analysis retrieves the kinetic constants describing the evolution of the system and the associated spectral components, the so-called EADS (evolution associated difference spectra) which are shown in Figure 4b. As it can be noticed, the evolution occurring on the 0.6 ps timescale, corresponding to the transition from the black to the red EADS, mainly consists in the rise of the positive band at short wavelengths and in a red-shift of both the excited state absorption band and of the stimulated emission band, which also partially recovers on this same timescale. The rise of an absorption band in the region where the *E*-isomer absorbs is indicative of the photoinduced *Z/E* isomerization event, which thus results to be an ultrafast process, as observed for other photoswitches – azobenzene in particular [32]. Upon this initial ultrafast evolution, the transient absorption signal intensity slightly decreases on the 7.5 ps timescale, as the result of vibrational relaxation, and mostly recovers on a ca. 140 ps timescale.

**Figure 5:**
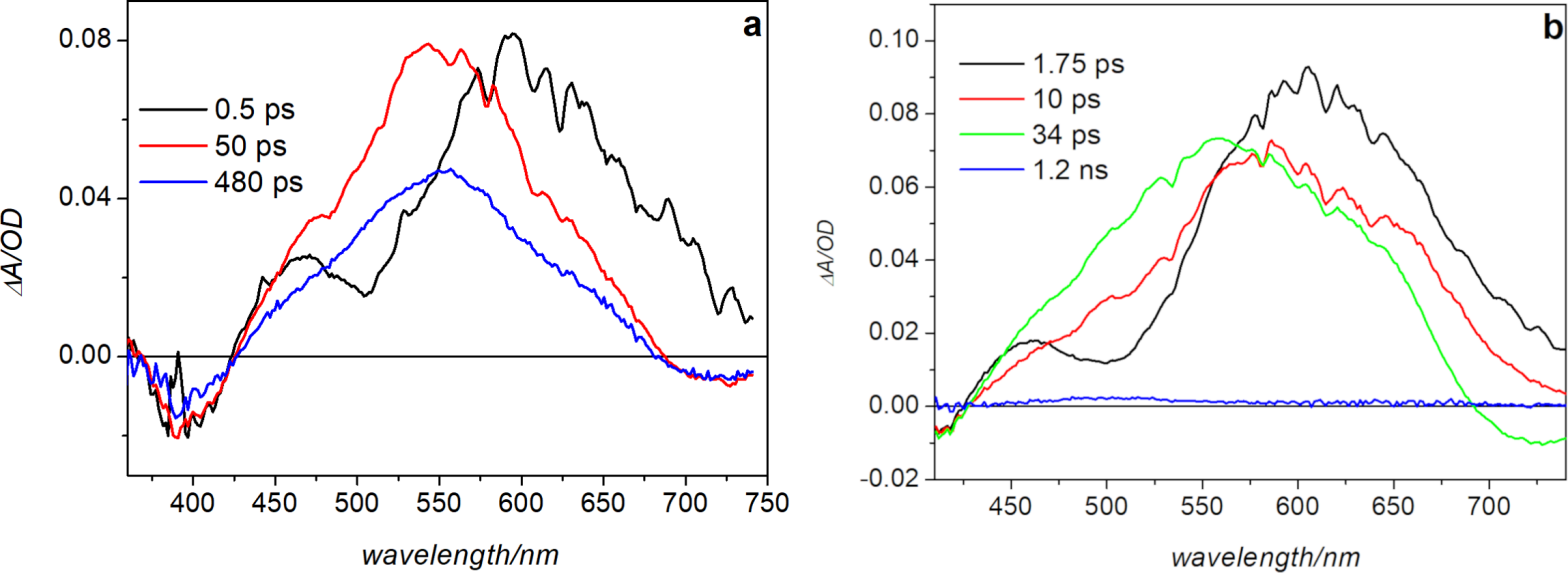
EADS obtained by global fit of the transient data recorded in a) acetonitrile and b) methanol upon excitation at 400 nm.

Transient absorption measurements upon 400 nm excitation were also performed in acetonitrile and methanol. While the *Z*-form is fluorescent in acetonitrile, the emission is strongly reduced in methanol [29]. The data were analyzed with the same global analysis procedure used for toluene, yielding the EADS shown in Figure 5.

**Figure 6:**
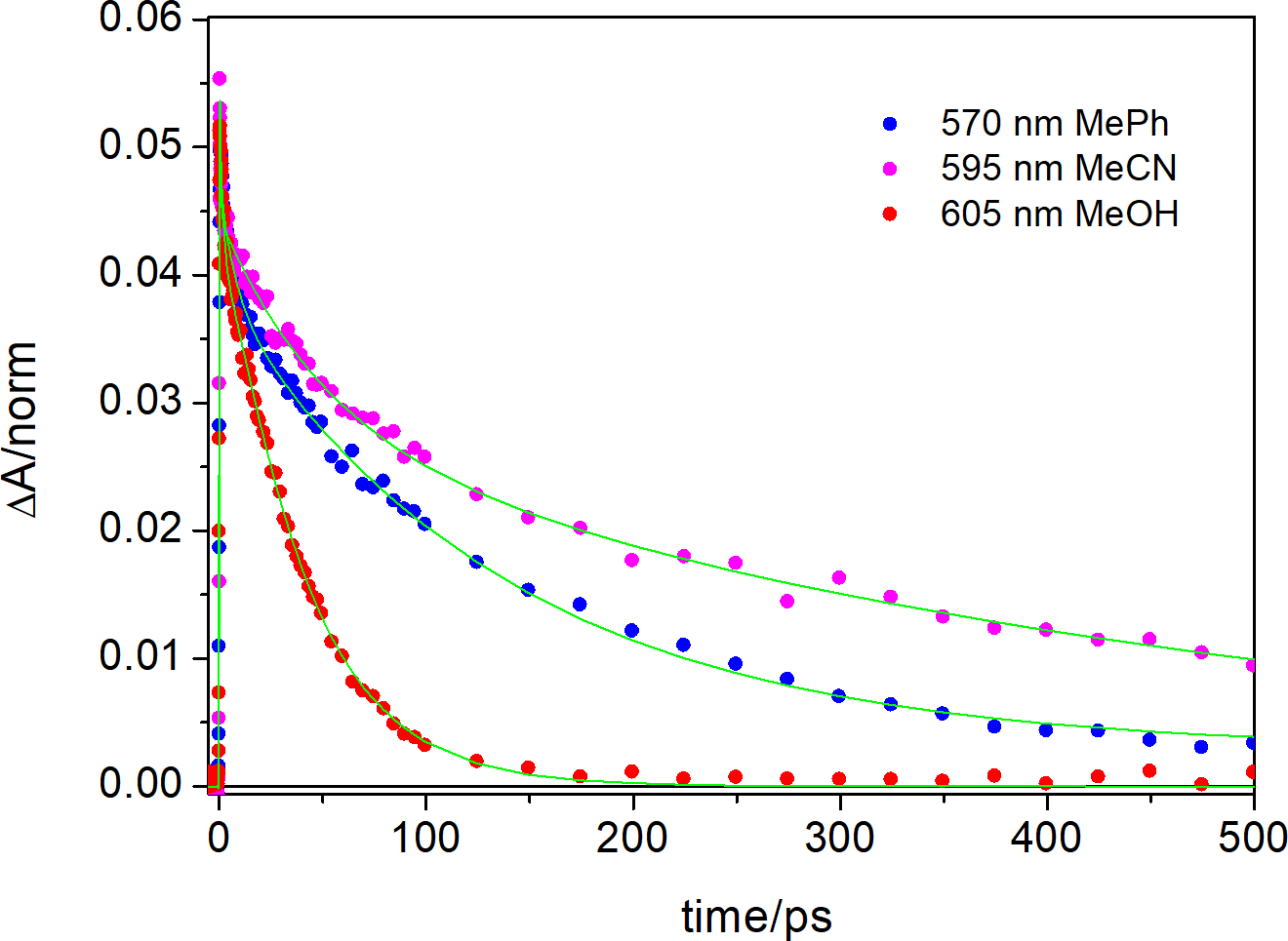
Kinetic traces recorded at the maximum of the excited state absorption band in toluene, acetonitrile and methanol.

The spectral evolution is very similar in these two solvents, although the time constants obtained by fitting the data are different. Due to lower solubility of the sample in the polar solvents, and increased scattering, data at short wavelengths are quite noisy in these measurements, especially in methanol, where a cutoff filter at 405 nm has been used during the measurement. Similarly, to what observed in toluene, at the short timescale the transient signal is characterized by a negative band peaked at about 400 nm, corresponding to ground state bleaching of the *Z*-form, an intense excited state absorption centered at about 600 nm, and a dip peaked at about 500 nm, as a result of stimulated emission; the latter is also observed in methanol. In acetonitrile, substantial spectral evolution is observed on a 0.5 ps timescale, mainly corresponding to the disappearance of the dip at about 500 nm and an overall blue shift of the positive signal (evolution from black to red EADS in Figure 5a). As previously observed in toluene, such an evolution, also associated to the rise of a positive band at <380 nm, whose observation is precluded due to scattering in polar solvents, reveals the occurrence of *Z*/*E* isomerization, whose kinetics is thus similar in toluene and acetonitrile. In the latter solvent, the signal intensity then decreases on a 50 ps timescale and recovers almost completely in about 500 ps. In methanol, although the spectral evolution is qualitatively similar to that observed in acetonitrile, the kinetics associated with the detected spectral changes differ substantially. The initial evolution, corresponding to the recovery of the dip at 500 nm and the decrease in intensity of the excited state absorption band centered around 600 nm, occurs on a longer timescale – about 1.8 ps, as compared to the sub-ps timescale observed both in acetonitrile and toluene. A blue shift of the excited state absorption band is then observed on a 10 ps timescale, and the almost complete recovery of the transient signal occurs in 34 ps. The very short excited state lifetime observed in methanol agrees with the low fluorescence in this solvent. Possibly, in the protic solvent the molecule adopts a less planar conformation, because of the competition between the intramolecular hydrogen bond between the N–H and C=O groups in the *Z*-form and hydrogen bonds formed by these functional groups with solvent molecules. The conformational distortion and the increased conformational disorder arising from hydrogen bonding with the solvent can be responsible for the fluorescence quenching in the *Z*-form. A comparison of the kinetic traces recorded on the maximum of the excited state absorption band in the three analyzed solvents (Figure 6) demonstrates that the excited state decay time decreases on going from acetonitrile to toluene, and further decreases in methanol. As shown in Supporting Information File 1 (Figure S3) the bleaching recovery of the not isomerized population fraction follows the same kinetics. The observed solvent dependence of the ESA decay could indicate that the excited state of the *Z*-form has a partial charge transfer nature, so that it can be stabilized in polar media where its lifetime slightly increases. In the protic solvent, however, where the molecule can adopt a partially twisted conformation, the increased conformational disorder most probably activates different non-radiative decay channels, shortening the excited state lifetime.

### Two-photon excitation

**Figure 7:**
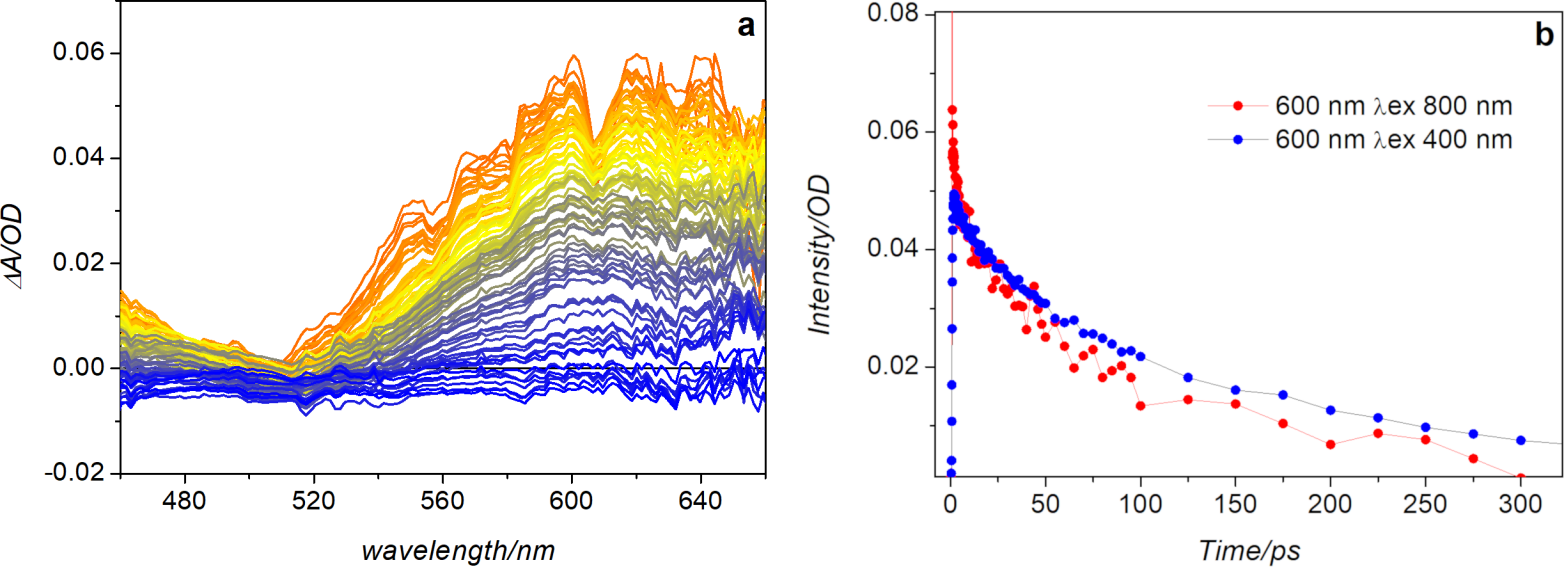
a) Transient absorption spectra measured for hydrazone **1** in toluene upon excitation at 785 nm. b) Comparison of the kinetic traces registered at 600 nm upon excitation at 400 nm and 785 nm.

Earlier experiments revealed that hydrazone **1** is able to isomerize also upon two-photon excitation [29]. In the present study we used transient absorption spectroscopy as an alternative method to determine the two-photon absorption cross section of the molecule, and compared the results with those previously obtained by exploiting the two-photon fluorescence of the system [29]. The transient spectra registered in toluene upon two-photon excitation, using a pump pulse at a 785 nm (Figure 7a), are qualitatively similar to those obtained upon 400 nm excitation (Figure 4), although the spectral region accessible to the probe is narrower than in the latter case. The bleaching signal is in fact covered as a consequence of the higher sample concentration needed to obtain a transient signal of sufficiently high intensity in order to get a good signal-to-noise ratio. Furthermore, the long wavelength region is strongly affected by the scattering of the excitation light, because of the quite intense pump pulse used for the experiment.

The comparison of the kinetic traces recorded at the maximum of the excited state absorption band upon one-photon and two-photon excitation, reported in Figure 7b, shows that the excited state evolution is similar in the two excitation conditions. A noticeable difference, however, is observed at short delay times after excitation, with a fast decay phase taking place for excitation at 785 nm. Nevertheless, the overall similarity of the successive evolution and of the transient spectra detected in the two cases indicates that a similar reactivity is induced upon one-photon and two-photon excitation, further confirming the two-photon photoswitching ability of hydrazone **1** already inferred by previous measurements [29].

Using a suitable standard, the transient absorption data recorded upon excitation at 785 nm allow for the determination of the two-photon absorption cross section, using the following expression [33]:

[1] $$\delta_{2}=\delta_{1}\left(\frac{\Delta A_{2}}{\Delta A_{1}}\right)\frac{\left(\sigma_{1}^{ex}-\sigma_{1}^{gr}\right)}{\left(\sigma_{2}^{ex}-\sigma_{2}^{gr}\right)}\frac{c_{1}}{c_{2}}$$

In this equation, index 1 and 2 refer respectively to the standard and the sample; Δ*A* is the absorbance at the maximum of the transient signal measured upon two-photon excitation, 
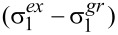
 is the difference in the absorption coefficient between the excited and ground state, which is retrieved by the one-photon transient absorption measurement, and *c* is the concentration of the sample. Taking a solution of coumarin 153 (δ_1_ = 47.4 GM [34]) as a reference, for which transient absorption spectra have been measured upon excitation at both 400 nm and 785 nm (see Figure S4, Supporting Information File 1), the estimated value for **1** in toluene is 12.6 GM, a value in good agreement with that previously determined through two-photon fluorescence measurements [29].

## Conclusion

Hydrazones are a promising new class of molecular photoswitches. In this work we investigated the spectroscopic properties of hydrazone **1**, a member of this family showing very interesting properties, such as high-isomerization quantum yield, fluorescence toggling and two-photon induced switching. Using a combination of time-resolved fluorescence and transient absorption spectroscopies we were able to gain new insights into the isomerization process of this molecule. Time-resolved luminescence measurements allowed us to determine the excited state lifetime of the *Z*-form, which strongly emits in the 500–520 nm spectral range. A fast component (1.3 ps in toluene) was also observed at shorter wavelengths and attributed to the *E*-isomer. The very short lifetime of this component accounts for the low emission quantum yield or even the absence of steady-state fluorescence of the *E*-isomers in most solvents. Transient absorption spectroscopy measurements, repeated in different solvents, allowed us to estimate the timescale of the *Z/E* isomerization process, which is about 0.5 ps in both toluene and acetonitrile, thus showing a negligible dependence on the solvent polarity. On the contrary, the excited state lifetime of the *Z*-isomer depends on the solvent properties and is especially short in MeOH, suggesting that the excited state of the molecule could have a partial charge transfer nature. Finally, transient absorption spectroscopy was employed as an alternative method to estimate the two-photon absorption cross section of hydrazone **1**, resulting in a value that is in very good agreement with the previous determination of this property, performed on the basis of fluorescence measurements [29].

## Materials and Methods

Hydrazone **1** was synthesized by following a previously reported procedure [29]. Its UV–vis spectra were recorded on a Shimadzu UV-1800 UV–vis spectrophotometer. A Photon Technology International QuantaMaster 4 spectrofluorometer outfitted with a LPS-100 lamp power supply and Xenon arc lamp housing, ASOC-10 electronics interface, MD-4 motor driver control, and a model 914D photomultiplier detector system were used to collect the fluorescence spectra of **1**.

### Time-resolved luminescence

Spectroscopic grade toluene from Merck was used as received. Solutions of **1** (4 × 10^−5^ M) in toluene were freshly prepared.

Time-resolved and spectral analysis of the fluorescence of the compound in the picosecond time regime were performed by means of a Hamamatsu synchroscan streak-camera apparatus (C10910-05 main unit and M10911-01 synchroscan unit) equipped with an ORCA-Flash 4.0 V2 charge-coupled device (CCD) and an Acton spectrograph SP2358. As excitation source a Newport Spectra Physics Solstice-F-1K-230 V laser system, combined with a TOPAS Prime (TPR-TOPAS-F) optical parametric amplifier (pulse width: 100 fs, 1 kHz repetition rate) [35] was used, tuned at 400 nm and 350 nm for predominant excitation of the *Z* and *E*-forms of **1**, respectively. To reduce photodegradation and limit the photoisomerization processes, the pump energy on the sample was reduced to 0.8 μJ/pulse at 400 nm and 4 μJ/pulse at 350 nm. Emission from the sample, collected at right angle with a 1 mm slit, was focused by means of a system of lenses into the spectrograph slit. Streak images were taken both in analog integration (200 exposures, 100 ms exposure time) and in photon counting (1000–10000 exposures, 20–30 ms exposure time). The decays were collected over emission spectral ranges of 20 nm. HPD-TA 9.3 software from Hamamatsu was used for data acquisition and analysis. The overall time resolution of the system after deconvolution procedure is 1 ps.

For fluorescence lifetime measurements in acetonitrile, solutions of **1** (1 × 10^−5^ M) were used. Fluorescence lifetime was determined by time-correlated single photon-counting (TCSPC) using a Photon Technology International QuantaMaster 4 spectrofluorometer integrated with Deltadiode-375L diode laser (λ_ex_ = 373 nm, <70 ps pulse width) as the excitation source. The fluorescence decays were detected using a fast PPD-850 detector. In all cases, decays were recorded until peak counts reached 10,000. The decay traces were analyzed by the one-exponential fitting method using Felix data analysis from Horiba Scientific Ltd.

### Transient absorption spectroscopy

The apparatus used for the transient absorption spectroscopy (TAS) measurements is based on a Ti:sapphire regenerative amplifier (BMI Alpha 1000) system pumped by a Ti:sapphire oscillator (Spectra Physics Tsunami). The system produces 100 fs pulses at 785 nm, 1 kHz repetition rate and average power of 450–500 mW. Excitation pulses at 400 nm have been obtained by second harmonic generation of the fundamental laser output. In case of two-photon excitation the fundamental beam at 785 nm has been directly employed as the pump. The pump beam polarization has been set to magic angle with respect to the probe beam by rotating a λ/2 plate. Excitation powers were on the order of 50–100 nJ for one-photon excitation and 1.7 μJ in case of two-photon excitation. The probe pulse was generated by focusing a small portion of the fundamental laser output radiation on a 2 mm thick calcium fluoride window. Pump-probe delays were introduced by sending the probe beam through a motorized stage. Multichannel detection was achieved by sending the white light continuum after passing through the sample to a flat field monochromator coupled to a home-made CCD detector. TAS measurements were carried out in a quartz cell (2 mm thick) mounted on a movable stage to avoid sample photodegradation and multiple photon excitation. The recorded kinetic traces and transient spectra have been analyzed by using a global analysis [30]. The number of kinetic components has been estimated by performing a preliminary singular values decomposition (SVD) analysis [31], global analysis was performed using the GLOTARAN package (http://glotaran.org) [36], and employing a linear unidirectional “sequential” model.

## Supporting Information

File 1 Additional spectra.
