# Supplementary material for: Ultrafast processes triggered by one- and two-photon excitation of a photochromic and luminescent hydrazone
Source: Beilstein J Org Chem. 2019 Oct 15;15:2438–46. doi: 10.3762/bjoc.15.236 (PMC6808199; doi:10.3762/bjoc.15.236)
Supplement: File 1 — Additional spectra. [file Beilstein_J_Org_Chem-15-2438-s001.pdf]

BEILSTEIN JOURNAL OF ORGANIC CHEMISTRY

Supporting Information

for

Ultrafast processes triggered by one- and two-photon excitation of a photochromic and luminescent hydrazone

Alessandro Iagatti, Baihao Shao, Alberto Credi, Barbara Ventura, Ivan Aprahamian and Mariangela Di Donato

Beilstein J. Org. Chem. **2019,** *15,* 2438–2446. doi:10.3762/bjoc.15.236

Additional spectra

License and Terms: This is a supporting information file under the terms of the Creative Commons Attribution License (http://creativecommons.org/licenses/by/4.0). Please note that the reuse, redistribution and reproduction in particular requires that the authors and source are credited.
The license is subject to the *Beilstein Journal of Organic Chemistry* terms and conditions: (https://www.beilstein-journals.org/bjoc)

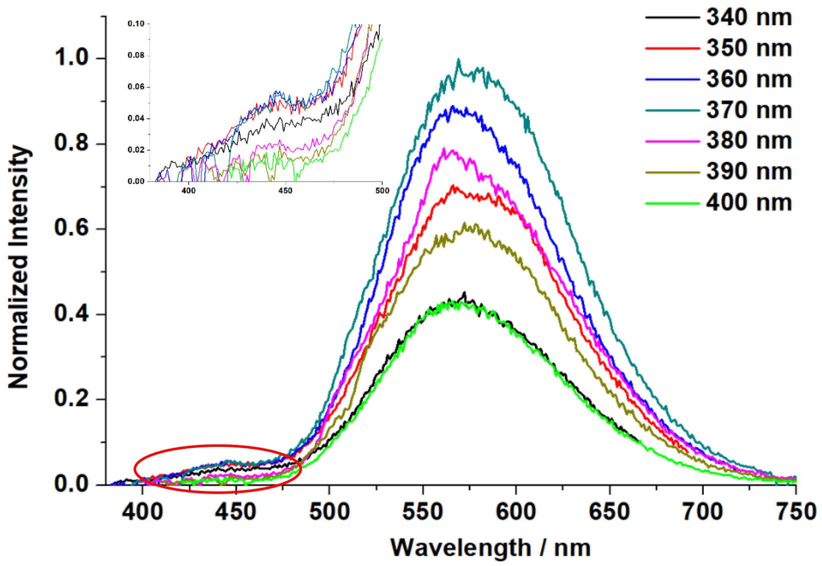

Figure S1: Fluorescence emission spectra of the *E*-rich solution of **1** (1×10^{-5} M; MeCN) upon excitation at various wavelengths (from 340 to 400 nm); The inset shows the zoom-in on the spectra between 380 and 500 nm (*E*-rich solutions were obtained upon irradiation at 442 nm to reach the PSS (**1**-*E* > 99%)).

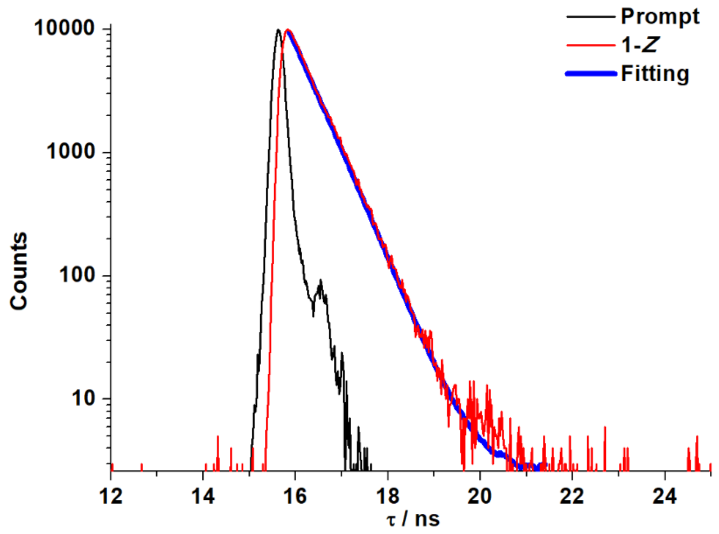

Figure S2: Fluorescence lifetime decay of **1-*Z*** (5×10^{-6}M) in acetonitrile. The fluorescence decay lifetime was determined to be 479 ± 3 ps based on three consecutive measurements.

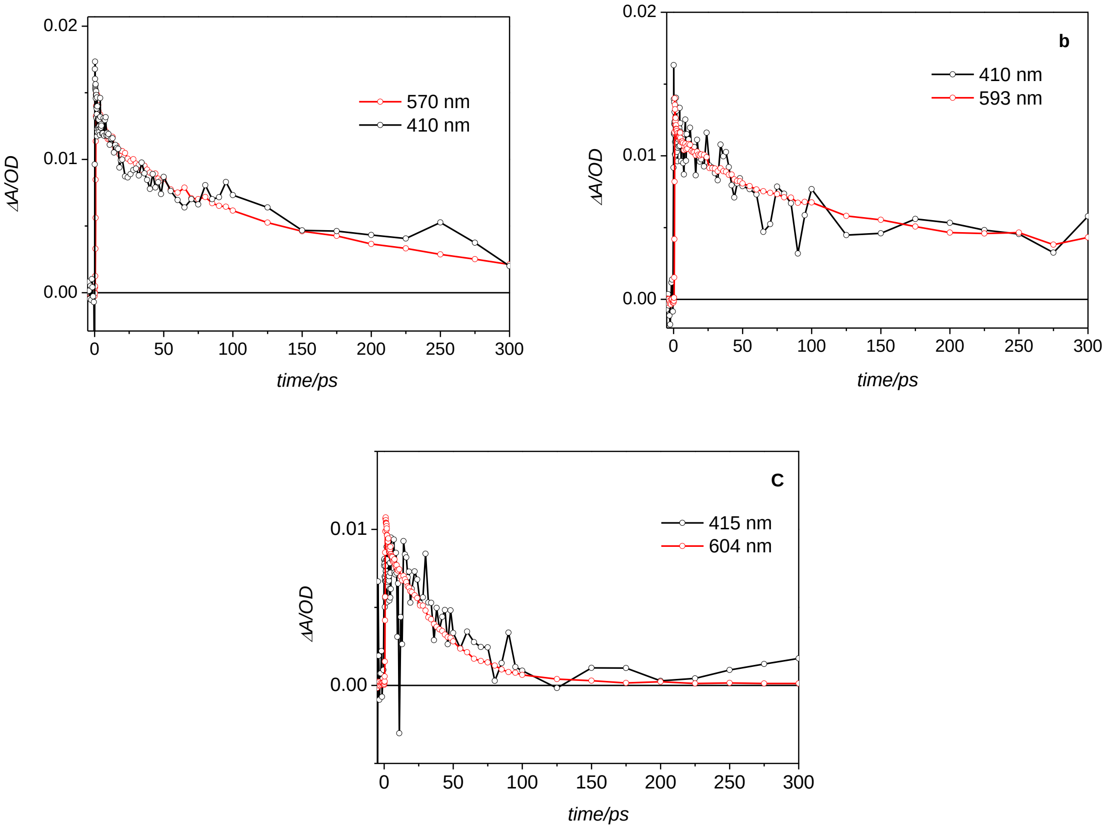

Figure S3: Comparison between the kinetics recorded in the bleaching (black) and ESA (red) region for a) toluene; b) acetonitrile; c) methanol. The sign of kinetic trace in the bleaching region has been inverted for comparison. The intensity of the ESA kinetics has been scaled as to match that of the bleaching one.

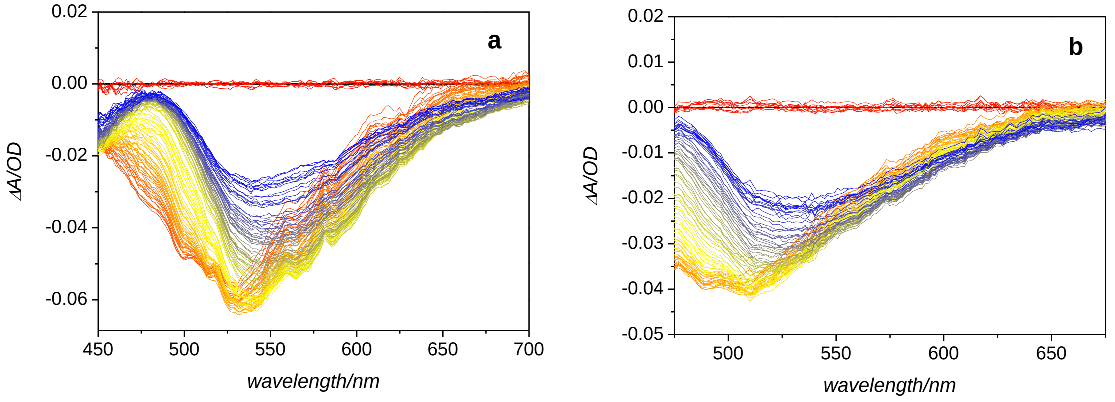

Figure S4: Transient absorption spectra of a solution of Cumarin 153 in ethanol registered upon a) one-photon excitation at 400 nm; b) two-photon excitation at 785 nm. Color code from red to blue indicates a timescale spanning from −5 ps to 1.5 ns.
